# Chemosensitizing effect of apigenin on T-ALL cell therapy

**DOI:** 10.3389/fphar.2025.1631505

**Published:** 2025-10-31

**Authors:** Nigar Huseynova, Züleyha Baran, Rovshan Khalilov, Afat Mammadova, Yusuf Baran

**Affiliations:** ^1^ Department of Biophysics and Biochemistry, Baku State University, Baku, Azerbaijan; ^2^ Department of Pharmacology, Laboratory of Molecular Pharmacology, Anadolu University, Eskişehir, Türkiye; ^3^ Department of Botany and Plant Physiology, Baku State University, Baku, Azerbaijan; ^4^ Department of Molecular Biology and Genetics, Laboratory of Cancer Genetics, İzmir Institute of Technology, İzmir, Türkiye

**Keywords:** apigenin, L-asparaginase, T-ALL, combination therapy, chemosensitization

## Abstract

T-cell acute lymphoblastic leukemia (T-ALL) is an aggressive hematological malignancy with limited therapeutic options and frequent treatment-associated toxicities. L-asparaginase, a cornerstone in T-ALL therapy, is often restricted by hypersensitivity reactions and systemic side effects, highlighting the need for safer strategies to enhance its efficacy. This study investigated the potential of apigenin, a naturally occurring flavonoid with antioxidant and pro-apoptotic properties, to act as a chemosensitizer for L-asparaginase in MOLT-4 T-ALL cells. Cytotoxicity was assessed using the MTT assay, apoptosis by Annexin V/PI staining, cell cycle distribution by flow cytometry, and mitochondrial membrane potential by JC-1 staining. Both apigenin and L-asparaginase produced dose- and time-dependent cytotoxicity, with combination treatment resulting in reduced IC_50_ values. Apoptotic analysis showed significantly higher apoptosis in the combination-treated groups than in single-agent groups. Cell cycle analysis revealed that apigenin induced S-phase arrest and L-asparaginase induced G1-phase arrest, while the combination disrupted cell cycle progression at multiple checkpoints. JC-1 assay further demonstrated enhanced mitochondrial depolarization, with up to a 29.2-fold increase in cytoplasmic-to-mitochondrial fluorescence ratio in combination therapy compared to L-asparaginase alone. These findings indicate that apigenin potentiates L-asparaginase-induced cytotoxicity through mitochondrial dysfunction and intrinsic apoptotic signaling. The combined use of apigenin and L-asparaginase may provide a novel strategy to improve therapeutic efficacy in T-ALL while potentially reducing the toxicity associated with high-dose L-asparaginase monotherapy.

## Introduction

Leukemia is a collection of cancers originating from abnormal cells in the body’s hematopoietic tissues, characterized by poor differentiation and aggressive behavior (Feng Li, 2024; Shafat et al., 2017; Yang et al., 2021). According to Sung et al., 2021, leukemia ranked as the 13th most common cause of cancer-related mortality worldwide, accounting for ∼3.1% (305,405 cases) of all cancer deaths. Among its subtypes, acute lymphoblastic leukemia (ALL) is a particularly aggressive form that arises from the lymphoid lineage, resulting in overproduction of immature lymphocytes and disruption of normal hematopoiesis. ALL is most prevalent in children, progresses rapidly, and requires prompt intervention (Ekpa et al., 2023; Pui et al., 2004; Rujkijyanont and Inaba, 2024).

T -cell acute lymphoblastic leukemia (T-ALL) is a rarer subtype, comprising 15%–20% of pediatric and 25%–30% of adult ALL cases, and is historically associated with inferior survival compared with B-ALL (Möricke et al., 2016; Raetz and Teachey, 2016). Despite improvements in chemotherapy protocols, outcomes for T-ALL, especially in relapsed and high-risk groups, remain poor (Durinck et al., 2015; Van Vlierberghe and Ferrando, 2012). The mainstay of treatment for ALL is combination chemotherapy that includes asparaginase, anthracyclines, cytarabine, cyclophosphamide, and intrathecal methotrexate (Hayashi et al., 2024; Juluri et al., 2022). While this regimen has improved survival to 80%–85%, management of resistant or recurrent disease is still challenging (Chen et al., 2013; Youns et al., 2010).

L-asparaginase is a crucial and highly effective drug for treating T-ALL (Egler et al., 2016; Ishida H, 2024; Tong and Rizzari, 2023). Its selective action is based on the inability of leukemic lymphoblasts to upregulate asparagine synthetase, leaving them vulnerable to extracellular asparagine depletion. However, dosing and administration are complicated by hypersensitivity, hepatotoxicity, coagulopathy, and pancreatitis, with hypersensitivity being the main cause of treatment interruption (Baruchel et al., 2020). Maintaining serum asparaginase activity (SAA) ≥0.1 IU/mL is required for therapeutic efficacy, but achieving this threshold while limiting toxicity is difficult (van der Sluis et al., 2016). Thus, strategies that enhance L-asparaginase efficacy while minimizing toxicity are urgently needed.

A major barrier in ALL therapy is the toxicity of chemotherapeutics to normal tissues, which restricts both dosing and treatment duration. Therefore, a promising approach is to combine conventional chemotherapy with natural, low-toxicity agents that enhance therapeutic efficacy while protecting healthy cells (Gilad et al., 2021). Plants are rich in bioactive compounds with anticancer potential, particularly polyphenols. These secondary metabolites influence multiple stages of carcinogenesis and are generally safe, affordable, and accessible (Kang et al., 2012; Kilani-Jaziri et al., 2012; Russo et al., 2010). Flavonoids, widely found in fruits, vegetables, teas, and herbal medicines, exhibit diverse pharmacological activities, including antioxidant, anti-inflammatory, hepatoprotective, immunoregulatory, and anticancer properties (Hasnat et al., 2024).

Among flavonoids, apigenin—a dietary flavone abundant in fruits, vegetables, and herbs—has attracted particular interest. It displays antioxidant, anti-inflammatory, and anticancer effects (Telange et al., 2017). Mechanistic studies show that apigenin arrests HL-60 myeloid leukemia cells at G2/M and TF-1 erythroid leukemia cells at G0/G1, partly through inhibition of the PI3K/AKT pathway and activation of caspases (A. Mahbub et al., 2017). Importantly, apigenin has low toxicity in normal cells, supporting its potential as an adjuvant to chemotherapy. It has also been shown to sensitize cancer cells to chemotherapeutic agents such as 5-fluorouracil, doxorubicin, chlorambucil, imatinib, and cyclophosphamide (Bokulić et al., 2011).

However, flavonoid–drug interactions can be context dependent. While many studies confirm their chemosensitizing potential, others report antagonistic effects. For example, apigenin has been shown to attenuate vincristine-induced apoptosis in hematological malignancy models (Goto et al., 2012). This variability highlights the need for rationally designed studies to define specific drug–flavonoid interactions in leukemia.

In this study, we investigated the potential of apigenin to sensitize T-ALL cells to L-asparaginase. By evaluating their combined effects on cell viability, apoptosis, mitochondrial function, and cell-cycle regulation, we aimed to identify a strategy to enhance L-asparaginase efficacy while reducing its dose-related toxicities, thereby improving therapeutic outcomes in T-ALL.

## Materials and methods

### Chemicals

L-Asparaginase from *E. coli* (A3809-1KU) and apigenin were purchased from Sigma (United States). A stock solution of L-asparaginase was prepared at 1 mg/mL using distilled water and stored at −20 °C. With dimethyl sulfoxide (DMSO), a stock solution of apigenin was made at a concentration of 2.5 mg/mL and stored at −20 °C.

The sterile DMSO was obtained from the Merck Group (Germany) and stored at room temperature. Fetal Bovine Serum (FBS) and RPMI 1640 media (1X) were sourced from Gibco (United Kingdom). Dulbecco’s Phosphate-buffered saline (PBS) (1X) was acquired from Capricorn Scientific (Germany), while Penicillin-Streptomycin (100X) was purchased from Euroclone (Italy). 3-(4,5-Dimethylthiazol-2-yl)-2,5-Diphenyltetrazolium Bromide (MTT) powder was obtained from Invitrogen (United States), and a stock solution was prepared in 1X PBS at a final concentration of 5 mg/mL. Trypan blue powder was obtained from Sigma Aldrich (United States), and a stock solution was prepared at a final concentration of 0.4% in 1X PBS. The FITC Annexin V Apoptosis Detection Kit I, used for apoptosis assays, was purchased from BD Biosciences (United States). The MitoProbe™ JC-1 Assay Kit for flow cytometry was acquired from Invitrogen (United States). Propidium iodide (PI) powder was sourced from AppliChem (Germany), and a 1 mg/mL stock solution was prepared in distilled water and stored at 4 °C. Triton X-100 was also purchased from AppliChem (Germany), while RNase A (DNase- and protease-free, 10 mg/mL) was obtained from Thermo Scientific (United States).

### Cell line

The human T-ALL cell line, MOLT-4 cells, was purchased from Icell Bioscience (Shanghai, China). The cell line used in this study was previously cryopreserved at −80 °C to maintain its viability and integrity before use. After thawing, cells were expanded under standard culture conditions, and experiments were performed using cells at the 3rd–4th passage to ensure stable growth and viability. The cells were grown in RPMI 1640 medium with 10% FBS, 1% L-glutamine, and 1% penicillin-streptomycin solution, and kept at 37 °C in a room with 5% CO_2_.

### Cell viability assay

Cell viability was determined using the 3-(4,5)-2,5-diphenyl-tetrazolium bromide (MTT) assay. MOLT-4 cells (1 × 10^4 cells per well) were placed in 96-well plates and given different amounts of L-asparaginase and apigenin, either by themselves or with a control, for 24 and 48 h at 37 °C in a 5% CO2 environment. At the conclusion of each incubation period, 20 µL of freshly prepared MTT solution (5 mg/mL) was added to each well and incubated for 4 h at 37 °C in a 5% CO_2_ environment. The absorbance was measured at 570 and 670 nm using a microplate reader (Thermo Scientific, Multiskan GO, Finland). The obtained absorbance values reflect cellular metabolic activity, which indirectly indicates viability and cytotoxicity. The values of IC25, IC50, and IC75 were determined based on the percentages of cell proliferation inhibition at different apigenin and L-asparaginase concentrations and were graphed.

### Apoptotic assay

MOLT-4 cells (6 × 10^5^ cells per well) were seeded into 6-well plates and exposed to varying concentrations of L-asparaginase (0.5, 1.0, and 1.5 μg/mL) and apigenin (5, 10, and 15 μg/mL), either individually or in combination, for 24 and 48 h at 37 °C in a 5% CO_2_ incubator. Apoptotic cells were evaluated using an Annexin V-FITC/propidium iodide (PI) apoptosis detection kit, following the manufacturer’s protocol. Quantification of apoptotic populations was performed using a flow cytometer (BD FACS Canto, United States) with two-channel analysis, and data were processed with CellQuest software (BD Biosciences).

### Determination of mitochondrial membrane potential

The MitoProbe™ JC-1 Assay Kit, which is used for flow cytometry, was used to check the mitochondrial transmembrane potential with a JC-1 dye test. MOLT-4 cells (6 × 10^5^ cells/well) were seeded into 6-well plates and were incubated with the indicated combined doses of apigenin and L-asparaginase (5μg/mL–0.5 μg/mL, 10μg/mL–1.0 μg/mL, and 15μg/mL–1.5 μg/mL) for 48 h in a 5% CO_2_ atmosphere. The experimental design included two untreated control groups and one negative control group, which contained cells exposed only to the solvent vehicles (distilled water and DMSO at their highest applied concentrations). After 48 h of incubation in a 5% CO_2_ atmosphere, carbonyl cyanide 3-chlorophenylhydrazone (CCCP; 2 μL, 50 mM), a compound that disrupts mitochondrial membrane potential, was added to one of the control groups to reach a final concentration of 50 μM and was maintained at 37 °C in a 5% CO_2_ atmosphere for 5 min. JC-1 dye (20 μL, 200 μM) was then added 20 min before the termination of the experiment and incubated at 37 °C in a 5% CO_2_ atmosphere. After incubation, the cells were collected and washed with PBS. Fluorescence intensity was ultimately measured using flow cytometry (BD FACS Canto, United States). In the JC-1 assay, P6 represents polarized mitochondria (healthy cells, red fluorescence), while P7 represents depolarized mitochondria (apoptotic cells, green fluorescence).

### Cell cycle analysis

MOLT-4 cells (6 × 10^5^ cells per well) were cultured in 6-well plates and exposed to various concentrations of apigenin and L-asparaginase, administered either separately or in combination. Two control groups were used: one group had cells treated with distilled water and DMSO at high levels, and the other group had cells that were not treated with any drugs at all. After 48 h of incubation in a 5% CO_2_ atmosphere, the cells were fixed with ethanol (80%) at −20 °C, then permeabilized with Triton X-100 in PBS (200 μL, 0.1%) and treated with RNase A (4 μL, 200 μg/mL) to remove RNA. Cells were stained with PI solution (20 μL, 1 mg/mL) and analyzed by flow cytometry (BD FACS Canto, United States). The percentage of cells in the G1, S, and G2 phases was measured to see how well apigenin and L-asparaginase, either separately or together, could stop the cell cycle by comparing the treated groups to the control groups.

### Isobologram and combination index analysis

The combination effects of L-asparaginase and apigenin were evaluated using the improved isobologram method and the combination index (CI) approach, as implemented in the CompuSyn software. Combination Index (CI) values were computed to evaluate the interaction between L-asparaginase and apigenin, with CI < 1 indicating a synergistic effect, CI = 1 representing an additive effect, and CI > 1 suggesting antagonism. These values were derived using the median-effect principle according to the Chou–Talalay method (Chou, 2010).

### Statistical analysis

Statistical analysis and graph generation were carried out using GraphPad Prism 10.0. A paired t-test was applied to assess differences between the control and experimental groups. Additionally, a two-way ANOVA was used to analyze the overall experimental data.

## Results

### Apigenin and L-asparaginase inhibit leukemia cell viability

**FIGURE 1:**
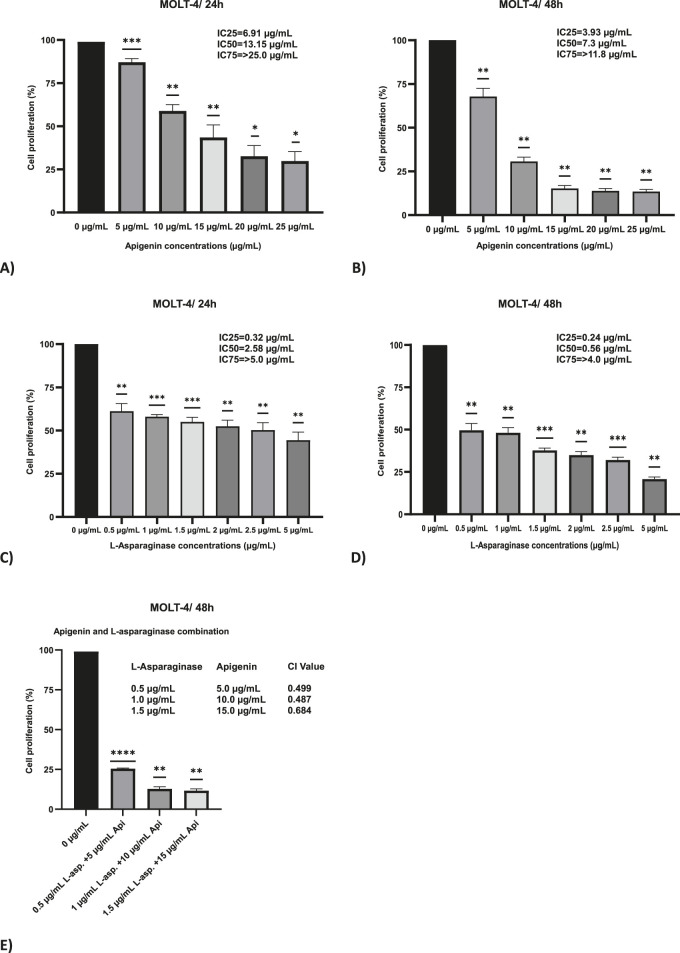
The effect of apigenin and L-asparaginase on the proliferation of MOLT-4 cells at 48 and 24 h. **(A)** Apigenin 24 h; **(B)** Apigenin 48 h; **(C)** L-asparaginase 24 h; **(D)** L-asparaginase 48 h; **(E)** Percentage proliferation data for apigenin + L-asparaginase combinations at 48 h. These data were used to calculate the Combination Index (CI), reported in the Results. Data represent mean ± SD of three independent experiments (*P < 0.01, **P < 0.05, vs. the control).

The MTT assay was employed to evaluate the cytotoxic effects of apigenin and L-asparaginase, both individually and in combination, on MOLT-4 cells following 24 and 48 h of incubation. For apigenin alone, there was a dose-dependent decrease in cell viability, which was stronger at 48 h than at 24 h (Figures 1A,B). The IC50 of apigenin was 13.15 μg/mL at 24 h but decreased to 7.3 μg/mL at 48 h, indicating that cytotoxic activity increased over time. Additionally, at 48 h, the IC25 and IC75 values were 3.93 μg/mL and 11.8 μg/mL, respectively, indicating a gradual dose-dependent response. Time-dependent cytotoxicity was evident at higher concentrations (≥15 μg/mL) of this compound.

An additional delay during the 48 h period further increased efficacy, with a dose-dependent inhibition of cell growth observed following treatment with L-asparaginase alone. The IC50 was 2.58 μg/mL at 24 h and decreased to 0.56 μg/mL at 48 h, representing a 4.6-fold reduction (Figures 1C,D). Moreover, at 48 h, the IC25 and IC75 values were 0.24 μg/mL and 4.0 μg/mL.

Combination index (CI) values below 1 at all tested concentrations, calculated using 48-h MTT assay viability data, indicated a synergistic interaction between apigenin and L-asparaginase. The CI values were 0.499 for the combination of 0.5 μg/mL L-asparaginase with 5 μg/mL apigenin, and 0.487 for 1 μg/mL L-asparaginase with 10 μg/mL apigenin, indicating strong synergy. At the highest tested dose—1.5 μg/mL L-asparaginase combined with 15 μg/mL apigenin—a slightly reduced synergistic effect was noted, with a CI value of 0.684 (Figure 1E).

### Cell cycle effects of L-asparaginase and/or apigenin in MOLT-4 cells

**FIGURE 2:**
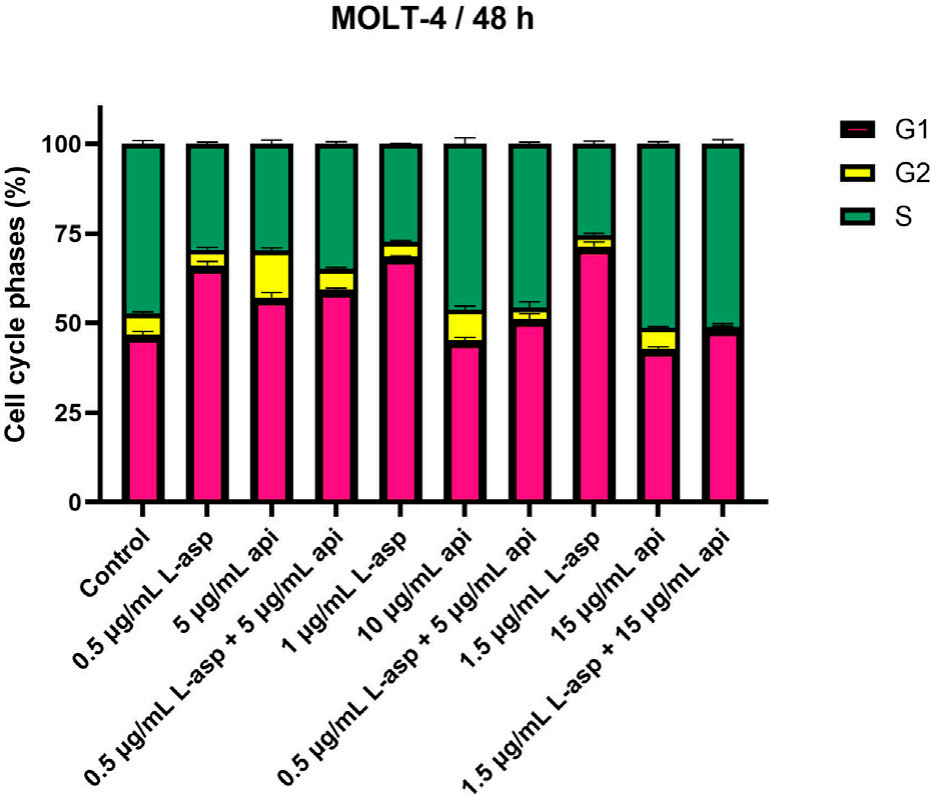
Apigenin and L-asparaginase induce cell-cycle arrest alone or in combination. After treatment with agents for 48 h, MOLT-4 cells were stained with PI and analyzed for cell cycle distribution using flow cytometry. Data represent mean ± SD of three independent experiments (*P < 0.01, **P < 0.05, vs. the control).

MOLT-4 cells were treated with apigenin and L-asparaginase alone and in combination for 48 h. The results suggested that either of the two compounds caused a concentration-dependent alteration of cell cycle progression, and at the cell cycle indices, they had a more prominent synergistic effect (Figure 2).

L-asparaginase treatment primarily led to G1-phase cell cycle arrest with a dose-dependent increase in the accumulation of cells in G1 phase. The percentage of G1 cells increased from 46.74% in the untreated control to 66.05% in the presence of 0.5 μg/mL. This effect was also more pronounced in higher concentrations, e.g., 1.5 μg/mL for 71.41% of cells in G1. As a result, the fraction of cells in S and G2 phases decreased. The S-phase cells decreased to 25.38% and the G2-phase cells were reduced to 3.22% at the 1.5 μg/mL value of L-asparaginase.

Contrasting results were obtained with apigenin treatment, where S phase arrest was shown in a higher percentage of cells at higher concentrations. The percentage of S-phase cells remained comparable to the control (47.35%), with 46.25% at 10 μg/mL, but increased further to 51.21% at 15 μg/mL. Conversely, G1-phase cells were reduced (15 μg/mL, 42.92%), and G2-phase cells were slightly reduced (15 μg/mL, 5.90%).

Using apigenin and L-asparaginase together at doses that were very effective in killing cells showed clear changes in how the cells progressed through their cycle. The number of cells in the G1 phase rose to 59.34%, while the number of cells in the S phase slowly dropped to 34.84% when treated with 0.5 μg/mL L-asparaginase and 5 μg/mL apigenin. G2 phase were also stable at 5.82%. Finally, the number of S-phase cells went up a lot to 45.62% compared to when 1 μg/mL L-asparaginase was used with 10 μg/mL apigenin; however, the number of G1-phase cells dropped to 51.19%. The G2 phase fell to 3.20%. The maximum extent of S-phase reached only 51.07% in L-asparaginase at 1.5 μg/mL and apigenin at 15 μg/mL, while the proportion of G1 phase cells was decreased down to 48.71% and G2 phase cells were almost absent, comprising only 0.23% of cells.

### Apoptotic assay

The apoptotic impact of apigenin and L-asparaginase, both individually and in combination, was assessed using Annexin V-FITC/PI dual staining followed by flow cytometry at 24- and 48-h post-treatment. Based on cytotoxicity results from the MTT assay, L-asparaginase was used at concentrations of 0.5, 1.0, and 1.5 μg/mL, while apigenin was applied at 5, 10, and 15 μg/mL. These same concentrations were combined to evaluate potential synergistic effects on apoptosis.

**FIGURE 3:**
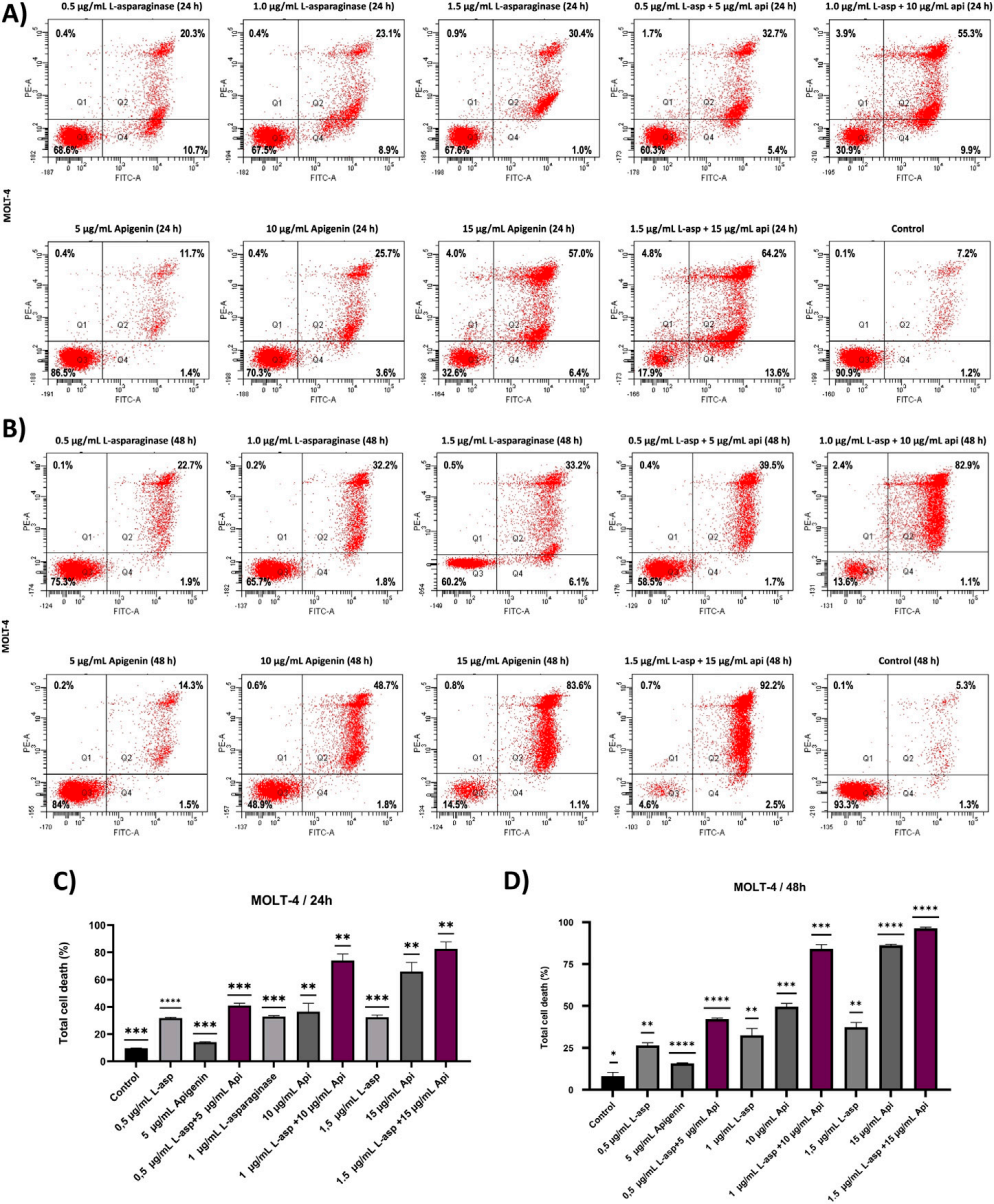
The apoptotic effect of apigenin and L-asparaginase alone and in combination on the proliferation of MOLT-4 cells at 24- and 48 h. **(A)** Apigenin and L-asparaginase alone and in combination at 24 h; **(B)** Apigenin and L-asparaginase alone and in combination at 48 h; **(C)** Quantification of total cell death in MOLT-4 cells treated with apigenin, L-asparaginase, or their combination for 24 h. **(D)** Quantification of total cell death in MOLT-4 cells treated with apigenin, L-asparaginase, or their combination for 48 h Data represent mean ± SD of three independent experiments (*P < 0.01, **P < 0.05, vs. the control).

The X-axis of the dot plots represents Annexin V-FITC staining, while the Y-axis denotes PI staining. Apoptotic cells were quantified as the sum of Q2 (late apoptosis) and Q4 (early apoptosis), while necrotic cells (Q1) were identified but not included in the apoptosis analysis (Figure 3A). Quadrant Q3 indicated the proportion of viable MOLT-4 cells, which was recorded as 90.9% at 24 h and 93.3% at 48 h in the control group. At 24 h, apigenin alone induced apoptosis in a dose-dependent manner, with 13.1%, 29.3%, and 63.4% apoptotic cells at 5, 10, and 15 μg/mL, respectively, while L-asparaginase alone caused 31.0%, 32.0%, and 31.4% apoptosis at 0.5, 1.0, and 1.5 μg/mL, respectively. Combination treatments significantly increased apoptotic activity, reaching 38.1% for 0.5 μg/mL L-asparaginase +5 μg/mL apigenin, 65.2% for 1.0 μg/mL + 10 μg/mL, and 77.8% for 1.5 μg/mL + 15 μg/mL, suggesting additive or synergistic interactions even at early time points.

After 48 h, apoptosis levels increased across all conditions: apigenin alone caused 15.8%, 50.5%, and 84.7% apoptosis at 5, 10, and 15 μg/mL, respectively, while L-asparaginase alone induced 24.6%, 34.0%, and 39.3% at 0.5, 1.0, and 1.5 μg/mL (Figure 3B). Notably, the combined treatments produced striking apoptosis rates of 41.2%, 84.0%, and 94.7% for 0.5 + 5, 1.0 + 10, and 1.5 + 15 μg/mL, respectively, reinforcing a robust time- and dose-dependent synergistic effect consistent with enhanced intrinsic apoptotic signaling and mitochondrial dysfunction as reported in similar studies (Aithal et al., 2019; Naponelli et al., 2024; W. Wang et al., 2000) The statistical analysis of three independently performed experiments is shown in Figures 3C,D, where the Y-axis represents the overall cell death rate calculated as the sum of Q1 (necrotic), Q2 (late apoptotic), and Q4 (early apoptotic) cell populations.

### Determination of mitochondrial membrane potential

**FIGURE 4:**
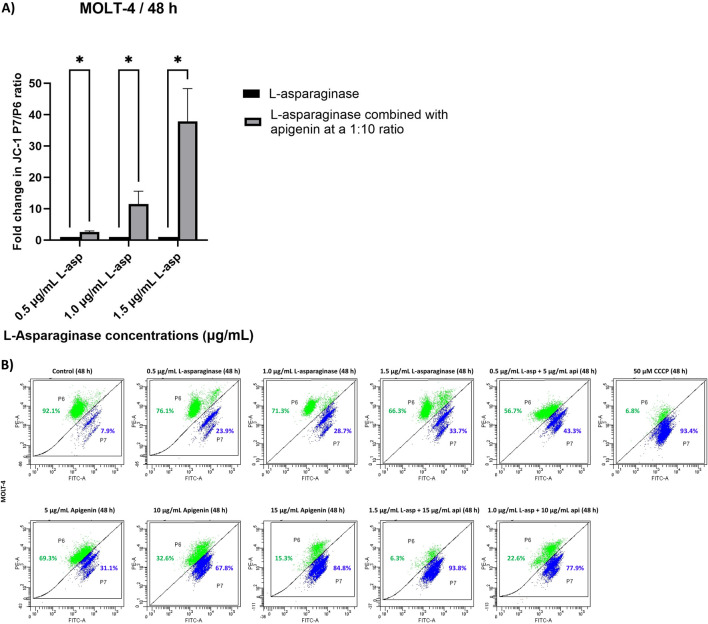
**(A)** Quantitative analysis of JC-1 red/green fluorescence ratio in MOLT-4 cells treated with L-asparaginase alone or in combination with apigenin for 48 h; **(B)** The effect of apigenin and L-asparaginase alone and in combination on the loss of mitochondria membrane potential of MOLT-4 cells at 48 h; Data represent mean ± SD of three independent experiments (*P < 0.01, **P < 0.05, vs. the control).

The mitochondrial membrane potential (ΔΨm) of MOLT-4 cells was evaluated after 48 h of treatment with apigenin and L-asparaginase, individually and in combination at different concentrations, using JC-1 dye-based flow cytometry. The percentages of P6 and P7 for each condition were reported alongside the results obtained from CCCP treatment (Figure 4B). CCCP, a well-established disruptor of mitochondrial membrane potential, was used to verify the sensitivity of the JC-1 dye in detecting changes in mitochondrial polarization in MOLT-4 cells. The data from the CCCP-treated group served as a reference for normalizing the values obtained from the untreated control samples. And drug-exposed groups. The calculated and normalized P7/P6 ratios of apigenin and L-asparaginase combination-treated groups were compared to the corresponding L-asparaginase-only groups to determine the relative fold change in the cytoplasmic/mitochondrial JC-1 ratio. This comparison was specifically chosen in accordance with the study’s aim to evaluate the chemosensitizing effect of apigenin on L-asparaginase-induced mitochondrial depolarization.

In comparison to the respective L-asparaginase-only groups (0.5, 1.0, and 1.5 μg/mL), the combination of 0.5 μg/mL L-asparaginase with 5 μg/mL apigenin produced only a modest change in the cytoplasmic-to-mitochondrial JC-1 ratio (2.4-fold increase over L-asparaginase alone). However, a substantial rise in this ratio was observed with the 1.0 μg/mL L-asparaginase +10 μg/mL apigenin combination (8.7-fold increase), and an even greater enhancement was recorded with the 1.5 μg/mL L-asparaginase +15 μg/mL apigenin treatment (29.2-fold increase relative to 1.5 μg/mL L-asparaginase) (Figure 4A).

## Discussion

Flavonoids are often mentioned to be able to sensitize malignant cells to classical anticancer drugs and potentiate their cytotoxicity, thus revealing their potential use as adjunctive agents for the treatment of neoplastic diseases including leukemia. It has been reported that flavonoids, specifically apigenin, can enhance the anticancer activity of chemotherapeutic agents (Mahbub et al., 2015; Mahbub et al., 2017; Mahbub et al., 2022). Consistent with these reports, our study demonstrates that apigenin augments the activity of L-asparaginase against T-ALL cells, supporting its role as a chemosensitizer.

Apigenin, a naturally occurring flavonoid, showed time-dependent inhibitory effects on leukemic cell viability, consistent with its reported ability to induce apoptosis through oxidative stress, mitochondrial membrane depolarization, and caspase activation (Rooprai et al., 2021; Shukla and Gupta, 2010). These findings also align with its recognized role as a chemosensitizing agent that enhances the responsiveness of cancer cells to therapy (Mahbub et al., 2022).

When combined, apigenin and L-asparaginase demonstrated clear synergistic interactions. This effect is likely driven by complementary mechanisms: L-asparaginase deprives cells of an essential amino acid, while apigenin lowers the apoptotic threshold by modulating mitochondrial pathways and apoptotic regulators. Together, these actions amplify cell death signals, as has been described for apigenin–flavonoid combinations in previous reports (B. Wang and Zhao, 2017).

Such synergy is particularly relevant in the clinical context, as it suggests the possibility of lowering effective L-asparaginase doses while maintaining efficacy, thereby reducing treatment-related toxicity. These observations are consistent with earlier findings that flavonoids, including apigenin, can potentiate the anticancer activity of standard chemotherapeutics (Asnaashari et al., 2023; Nozhat et al., 2021).

Cell-cycle analyses further highlight the complementary actions of both agents. L-asparaginase primarily induced G1 arrest, consistent with its role in limiting asparagine availability essential for DNA replication and protein synthesis (Takahashi et al., 2017) whereas apigenin promoted S-phase accumulation, a phenomenon linked to oxidative stress–mediated DNA damage and inhibition of cyclin-dependent regulators (Shi et al., 2015). Their combination produced mixed G1 and S-phase arrest, suggesting a dual blockade at multiple checkpoints. Such dual-phase interference may prevent adaptive resistance, a frequent obstacle in leukemia therapy (Ghelli Luserna di Rora’ et al., 2017; Simabuco et al., 2018).

Apoptosis assays further support the hypothesis that apigenin enhances L-asparaginase efficacy. Mechanistically, flavonoids such as apigenin are known to destabilize mitochondrial membranes, upregulate pro-apoptotic proteins, and inhibit survival pathways including PI3K/AKT and mTOR, thereby amplifying intrinsic apoptotic signaling (Naponelli et al., 2024; Zughaibi et al., 2021). The strong apoptotic responses in the combination groups are consistent with these mechanisms, underscoring apigenin’s role as a chemosensitizer.

Interestingly, while L-asparaginase alone maintained relatively consistent apoptotic activity over time, apigenin’s effects were highly dose- and time-responsive, reinforcing its role as a dynamic enhancer of cell death when used in combination. The combination’s superiority was further substantiated by the inclusion of both early and late apoptotic events (Q4 and Q2), while excluding necrosis (Q1), ensuring that the measured responses specifically reflect programmed cell death.

Altogether, these results suggest that co-administration of apigenin may allow for the use of lower L-asparaginase doses while maintaining or enhancing therapeutic efficacy, which could potentially minimize L-asparaginase-associated toxicity in clinical applications.

The mitochondrial depolarization observed with JC-1 staining further reinforces this interpretation. While L-asparaginase alone induces apoptosis largely through ER stress and protein synthesis inhibition (Hawkins et al., 2004), apigenin directly targets mitochondria by modulating Bax/Bcl-2 balance and cytochrome c release (Çetinkaya and Baran, 2023; Huseynova et al., 2024). The pronounced mitochondrial dysfunction in the combination groups reflects a convergence of these mechanisms, leading to amplified intrinsic apoptosis.

Taken together, these findings suggest that apigenin enhances the therapeutic potential of L-asparaginase in T-ALL cells by acting on complementary pathways involving cell-cycle arrest and mitochondrial-mediated apoptosis. By enabling dose reduction of L-asparaginase without loss of efficacy, this strategy could help overcome toxicity-related limitations in clinical settings. Future work should focus on elucidating the precise molecular targets of this synergy and validating these effects *in vivo* to assess translational potential.

## Conclusion

This study highlights the potential of combining natural flavonoids with conventional chemotherapeutic agents to enhance anticancer efficacy. Specifically, the flavonoid apigenin significantly potentiated the cytotoxic, apoptotic, and mitochondrial-disrupting effects of L-asparaginase in leukemic cells. The combination treatment led to greater reductions in cell viability, increased rates of programmed cell death, and enhanced mitochondrial membrane depolarization compared to either agent alone, suggesting a synergistic interaction.

Mechanistically, the results suggest that apigenin may sensitize cancer cells to chemotherapy by modulating mitochondrial function and apoptotic signaling pathways. Additionally, distinct cell cycle arrest patterns induced by each agent contributed to their combined effectiveness, potentially limiting cancer cell adaptability and resistance.

These findings support the growing interest in using plant-derived bioactive compounds as adjuvants in cancer therapy. The observed synergy between apigenin and L-asparaginase provides a promising foundation for future research and highlights the potential for reduced dosing and improved therapeutic outcomes in leukemia and possibly other malignancies. Further studies, including *in vivo* models and clinical evaluation, are warranted to fully explore and validate this combination strategy.

## Data Availability

The original contributions presented in the study are included in the article/supplementary material, further inquiries can be directed to the corresponding authors.

## Abbreviations

ALL, Acute Lymphoblastic Leukemia; ANOVA, Analysis of Variance; ATCC, American Type Culture Collection (inferred from context, not explicitly stated but common for cell lines); B-ALL, B-cell Acute Lymphoblastic Leukemia; CDK2, Cyclin-dependent Kinase 2; CCCP, Carbonyl Cyanide 3-Chlorophenylhydrazone; CI, Combination Index; DMSO, Dimethyl Sulfoxide; DNA, Deoxyribonucleic Acid; ER, Endoplasmic Reticulum; FACS, Fluorescence-Activated Cell Sorting; FBS, Fetal Bovine Serum; FITC, Fluorescein Isothiocyanate; G1, Gap 1 Phase; G2, Gap 2 Phase; JC-1, 5,5′,6,6′-Tetrachloro-1,1′,3,3′-tetraethylbenzimidazolylcarbocyanine iodide; MMP, Mitochondrial Membrane Potential (appears as ΔΨm); MTT, 3-(4,5-Dimethylthiazol-2-yl)-2,5-Diphenyltetrazolium Bromide; PBS, Phosphate-Buffered Saline; PI, Propidium Iodide; PKB, Protein Kinase B (another name for AKT); PMSF, Phenylmethylsulfonyl Fluoride (not mentioned, but often used with protease-free conditions—could be excluded if not present); qPCR, Quantitative Polymerase Chain Reaction (not in your document, but often associated with gene expression studies—omit if not present); RNA, Ribonucleic Acid; RNase, Ribonuclease; SAA, Serum Asparaginase Activity; SDS, Sodium Dodecyl Sulfate (not used in your text); SD, Standard Deviation; T-ALL, T-cell Acute Lymphoblastic Leukemia; ΔΨm, Mitochondrial Membrane Potential.
